# Executive function and language: a behavioural and functional near-infrared spectroscopy study in 23 normally hearing children and one deaf child with cochlear implants

**DOI:** 10.3389/fnhum.2026.1719052

**Published:** 2026-04-08

**Authors:** Rachael J. Lawrence, Efstratia Papoutselou, Guangting Mai, Ian M. Wiggins, Douglas E. H. Hartley

**Affiliations:** 1 Nottingham Biomedical Research Centre, National Institute for Health Research (NIHR), Nottingham, United Kingdom; 2 Hearing Sciences, Division of Mental Health and Clinical Neuroscience, School of Medicine, University of Nottingham, Nottingham, United Kingdom; 3 Nottingham University Hospitals NHS Trust, Nottingham, United Kingdom

**Keywords:** children, cochlear implants, executive function, fNIRS, functional near-infrared spectroscopy, language processing, neuroimaging

## Abstract

**Introduction:**

Emerging evidence suggests that neurocognitive processes, notably executive function (EF), should be considered important contributors to the observed variability in language outcome in both normally hearing (NH) and deaf children. Neural correlates of EF have potential clinical value, especially for infants and young children, in whom behavioural assessments can be unreliable.

**Methods:**

In the current study, we aimed to examine cortical correlates of EF in NH children aged 4–6 years old using functional near-infrared spectroscopy (fNIRS), a non-invasive, neuroimaging technique. In 23 NH children we measured activity in superior temporal and prefrontal cortex bilaterally whilst participants performed a behavioural EF task.

**Results:**

A significant association between the amplitude of EF-evoked cortical activation overlying the left dorsolateral prefrontal cortex and language performance was observed in NH children. To demonstrate that fNIRS is feasibly capable of measuring cortical responses specifically to behavioural EF in paediatric cochlear implant (CI) recipients, we also present the data for one deaf child aged 4.3 years with (CIs) who underwent the same simultaneous behavioural EF tasks and fNIRS imaging as the NH children.

**Discussion:**

The current results not only highlight the importance of higher-order top-down EF processes in language development, but they also demonstrate potential for fNIRS to provide cortical correlates of EF performance. Future applications of this technology could not only help explain variability in language outcome in both NH children and deaf children with CIs but also facilitate earlier intervention, such as EF rehabilitation strategies, where required at an earlier stage in a child’s life.

## 1 Introduction

Executive function describes the cognitive processes required to achieve planned and goal-directed actions (Diamond, 2013) and is generally accepted to consist of three main elements: (1) working memory (WM), temporarily holding and processing information in immediate memory to guide reasoning and decision-making; (2) inhibition control (IC), the ability to suppress specific thoughts or behaviour and; (3) cognitive flexibility (CF), the ability to switch between a mental or behavioural task in response to changing demands (Miyake et al., 2000). Despite not being described in Miyake et al. (2000) model, many researchers consider attention to be fundamental to EF development (Garon et al., 2008). In the typically developing child, EF begins to develop during infancy with rapid changes occurring during the preschool years that continue to develop through adolescence and into adulthood (Anderson, 2002; Espy et al., 2001; Zelazo et al., 2013). An association between EF and language processing is well established (Fuhs and Day, 2011; Kuhn et al., 2016), with the current literature commonly describing a reciprocal and bidirectional relationship between EF and language in both NH and hearing impaired children, particularly in early childhood (Kronenberger et al., 2020; Singer and Bashir, 1999). Executive function also regulates listening effort that, subsequently, increases listening proficiency (Kronenberger et al., 2020). This increased listening proficiency is proposed to allow for more effective language processing, an important requirement for deaf children with CIs who need to encode the degraded input from their hearing device (Pichora-Fuller et al., 2016).

A plethora of neuroimaging studies in children with typical brain development demonstrate a relationship between behavioural measures of EF and activity within the prefrontal cortex (PFC) (Aron et al., 2014; Konishi et al., 1998; Laird et al., 2005; Monchi et al., 2001; Wager and Smith, 2003). With respect to the auditory-deprived brain, evidence suggests that congenital deafness affects the development of neuronal connections between primary auditory (i.e., superior temporal cortex) and higher-order cortical areas such as the PFC (Sharma et al., 2009). Since both prefrontal and temporal brain regions are reportedly involved in WM processing (Baddeley, 2003), it is perhaps not that surprising that pre-lingually deaf children with CIs are two-to-five-times more likely than NH children to have deficient EF skills (Kronenberger et al., 2014a). Variable performance in EF has been observed in paediatric CI recipients (who are encouraged to use oral rather than visual/sign language) as early as during the preschool years (Beer et al., 2014), a period during which the development of EF and language skills are potentially related (Pichora-Fuller et al., 2016). This evidence contributes to the hypothesis that the observed variability in language outcome amongst paediatric CI recipients may be accounted for by differences in neurocognitive processing (Conway et al., 2011; Geers et al., 2011). Interestingly however, a study by Hall et al. (2018) showed that deaf native signers who had access to American Sign Language from birth showed no evidence of problems with performance-related EF compared with NH children. This suggests that deafness itself does not account for poor EF, rather a lack of early access to language whether that be in sign or speech, has a stronger impact. Hence, when analysing the relationship between EF and language development in deaf children, the mode of communication is a vital factor. For deaf children who have received a cochlear implant, individual communication experiences both pre- and post-implantation need to be considered.

EF in preschool children with NH has been shown to improve with behavioural interventions (Blair and Raver, 2014; Diamond and Lee, 2011), hence there may be value in integrating such treatments into existing speech and language therapy regimens for children with and without hearing loss (Kronenberger et al., 2020). Currently, assessments of EF abilities are reliant on behavioural measures. However, since very young children are often difficult to assess with behavioural techniques, there is often a delay of many years before it is possible to detect impaired EF development that delays any subsequent treatment.

Functional near-infrared spectroscopy (fNIRS) is an increasingly popular, non-invasive optical imaging technique that can be used safely and repeatedly for studying cortical function (Saliba et al., 2016). It has many advantages over other neuroimaging techniques, which include being: (1) acoustically silent; (2) portable, allowing for subjects to be imaged in both clinical and research environments; (3) relatively resistant to head movements so that infants and children can be scanned whilst awake and sitting on a parent’s knee; (4) compatible with hearing devices, including CIs. Indeed, in NH individuals and deaf CI users alike, fNIRS has reliably measured cortical responses in the paediatric population (Blasi et al., 2014; Lawrence et al., 2020; Mushtaq et al., 2020; Sevy et al., 2010). fNIRS images the haemodynamic response to neuronal activity in the brain via the use of near-infrared light (Boas et al., 2014). Low-power near-infrared light is directed through the scalp and into the cortex; the intensity of the light returning to the surface of the scalp is then detected. Changes in the concentration of oxygenated haemoglobin (HbO) and deoxygenated haemoglobin (HbR) can be measured, which are then subsequently interpreted as an indirect reflection of neuronal activity.

The overall aim of this work was to evaluate whether behavioural EF task-evoked cortical activation correlates with language performance in NH children aged 4–6 years. We use fNIRS imaging to principally target prefrontal and superior temporal brain regions in NH children to examine cortical activation during visual WM and IC behavioural EF tasks. We also include one deaf child with CIs to evaluate whether fNIRS is capable of successfully measuring cortical responses, specifically to behavioural EF, in paediatric CI recipients. It was our understanding at the time of conducting the current study, that although fNIRS had been utilised to examine behvaioural EF-evoked cortical activation in NH children (Smith et al., 2017; Mehnert et al., 2013), it had never previously been used to investigate cortical responses to behavioural EF in children with CIs. Where comparisons are made between NH children and one deaf child with CIs, this is on a qualitative and descriptive case study type basis only.

## 2 Materials and methods

### 2.1 Participants and ethical approval

The design was approved by the Health and Social Care Research Ethics Committee A (HSC RECA) (REC reference: 19/NI/0121) and sponsored by Nottingham University Hospitals NHS Trust (Research & Innovation reference: 19ET008). Written informed consent was obtained from the accompanying parents or guardians of all participants. All NH participants were native English speakers, able to talk in simple sentences, had normal or corrected-to-normal vision and were aged between 4 years, 0 months and 6 years, 11 months. Any known cognitive, motor, or language disorder were defined as exclusion criteria. Children were recruited into the NH group if their parent/guardian declared no known problems with their hearing. The deaf child had been diagnosed with a bilateral profound hearing loss at 2 years of age and underwent bilateral cochlear implantation at 3 years of age. With respect to communication mode, the deaf participant was born into a NH family and was encouraged to develop spoken English language from birth with no regular exposure to visual/sign language. Although a formal cognitive assessment was not performed, there was no known cognitive or medical disorders apart from profound sensorineural deafness.

### 2.2 Study design

Twenty-four NH children were recruited to this experiment, with a mean age of 5.3 years (range 4.0–6.8 years, [SD] = 0.8 years). Table 1 details individual ages of the recruited participants. One deaf child with CIs was also recruited (male, aged 4.3 years). However, one NH child refused to wear the fNIRS equipment during the Go/NoGo behavioural experiment and refused to perform the N-back task. Therefore, cortical activation data acquired from 23 NH participants and one deaf child with CIs was subjected to analysis. Behavioural EF data was acquired for all 24 NH children and the deaf children with CIs, except for the one NH child who refused to perform the N-back task.

**Table 1:** A table to demonstrate the individual age of the 24 NH participants.

Participant ID	Age in years
1	4.4
2	5.4
3	4.2
4	5.3
5	4.8
6	4.9
7	6.3
8	4.0
9	6.0
10	5.9
11	4.6
12	6.8
13	4.9
14	6.0
15	6.0
16	6.2
17	5.2
18	5.0
19	5.6
20	5.4
21	6.0
22	4.8
23	4.4
24	5.8

Participant 1 was excluded from fNIRS analysis since they refused to perform the N-back task and wear the fNIRS equipment during the Go/NoGo task. N.B. Mean age 5.3 years (SD 0.8).

#### 2.2.1 Equipment

Testing was conducted in a sound-attenuated room with the lighting dimmed. Participants were seated approximately 75 cm from a visual display unit. Brain activity was non-invasively measured using a Hitachi (Tokyo, Japan) ETG-4000 continuous-wave fNIRS system. The ETG-4000 measures simultaneously at wavelengths of 695 nm and 830 nm (sampling rate 10 Hz) and uses frequency modulation to minimise crosstalk between channels and wavelengths (Scholkmann et al., 2014). A dense sound-absorbing screen was placed between the fNIRS equipment and the listening position, resulting in a steady ambient noise level of 38 dB SPL (A-weighted). During the main fNIRS task, participants entered their responses using an “RTbox” button box (Li et al., 2010). The experiment was implemented in MATLAB (MathWorks, Natick, MA, USA) using the Psychtoolbox-3 extensions (Brainard, 1997; Pelli, 1997).

#### 2.2.2 Behavioural stimuli

The Go/NoGo and N-back tasks were employed to examine behavioural EF-evoked fNIRS responses since these tasks have been commonly employed in neuroimaging studies examining IC and visual WM (Smith et al., 2017). The methodology and stimulation paradigms for the Go/NoGo (visual IC task) and N-back (visual WM) tasks that were utilised in the Smith et al. (2017) fNIRS study were implemented in current experiments.

##### 2.2.2.1 Go/NoGo behavioural task

**Figure 1:**
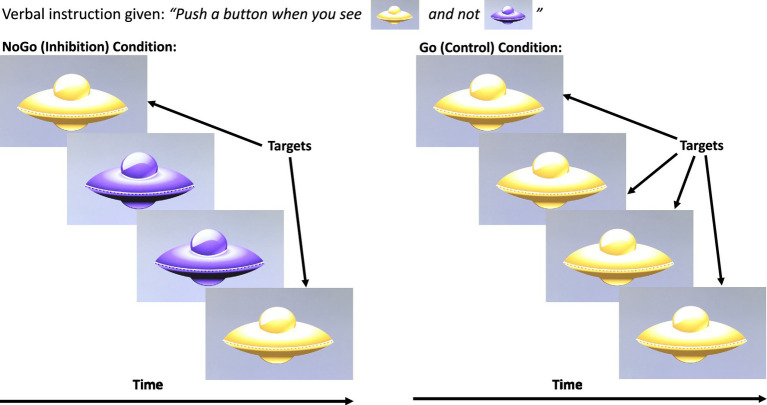
The response inhibition control Go/No-Go task. Adapted with permission from Smith et al. (2017).

In this task, yellow and purple spaceships appeared sequentially on the visual display unit (VDU). Participants were instructed to press a button on the “RTbox” response box when they saw a yellow spaceship, and not when they saw a purple spaceship (Figure 1). A mandatory practice session with both yellow and purple spaceships was completed and repeated, if necessary, until the child attained a score of ≥70% correct. Subsequently, each participant was presented with four Go blocks (control condition) alternating with four NoGo blocks (inhibition condition) with 10 s of rest between each block. The Go blocks included only yellow spaceships, accounting for basic visual processing and motor response. During the rest block a fixation cross was presented on a uniform background. The NoGo blocks included both yellow and purple spaceships at a ratio of 10:4. For all blocks, 10 spaceships appeared for 500 milliseconds (ms) with an inter-stimulus interval of 1,000 ms, for a total of 15 s per block, with 10 s of rest between blocks.

##### 2.2.2.2 N-back behavioural task

**Figure 2:**
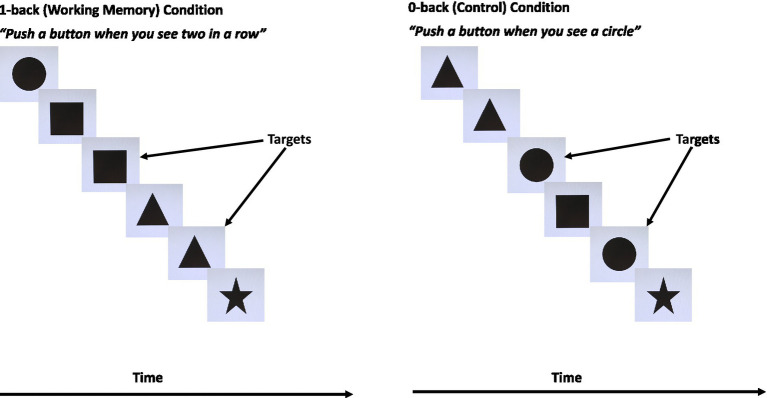
The working memory N-back task. Adapted with permission from Smith et al. (2017).

The N-back task consisted of a WM condition (1-back) and a control condition (0-back). For the 1-back condition, various black shapes (square, circle, triangle, star) on a uniform background were presented to participants who were instructed to push a button if they saw the same shape twice in a row (Figure 2). Stimuli were presented for 1,500 ms each with an inter-stimulus interval of 500 ms. Practice blocks were completed until a score of ≥70% correct was obtained. The participant then completed four blocks of 16 s each with a target/non-target ratio of 3:5. During the 0-back blocks, participants were told to press a button whenever they saw a circle (an example circle was shown prior to onset of the block). Four 0-back blocks were completed, each block lasting 16 s with a target/non-target ratio of 3:5. A 10 s rest period occurred between all blocks.

As per Smith et al. (2017), behavioural analyses for the Go/NoGo and N-back tasks involved calculating task accuracy. Overall task accuracy was determined using d’prime, which was calculated using the following formula:


$$d^{'}\text{prime}(p_{i})=z(\text{omission}\;{\text{error}}_{i})–z(\text{comission}\;{\text{error rate}}_{i})$$

where z is the z score for the ith participant, omission error rate (OER) represents not pressing the response button when seeing a yellow spaceship and commission error rate (CER) reflects pressing when seeing a purple spaceship. d’prime is known to represent task accuracy while accounting for different response styles (Macmillan and Creelman, 2004).

#### 2.2.3 fNIRS task procedure

The study design used was based on previous studies performed in our laboratory (Lawrence et al., 2018; Lawrence et al., 2020; Wijayasiri et al., 2017) by simultaneously performing fNIRS imaging along with the behavioural tasks.

#### 2.2.4 fNIRS measurements and definition of regions of interest (ROIs)

fNIRS measurements were made with a total of 33 optodes arranged in a 3 × 11 array within an elasticated fabric head cap. The array comprised 17 emitter and 16 detector optodes with a fixed inter-optode distance of 30 mm, providing a penetration depth into the brain of approximately 15 mm (Gibson et al., 2005; Strangman et al., 2014). This resulted in a total of 52 measurement channels, primarily covering the prefrontal and superior temporal cortex (STC) bilaterally.

**Figure 3:**
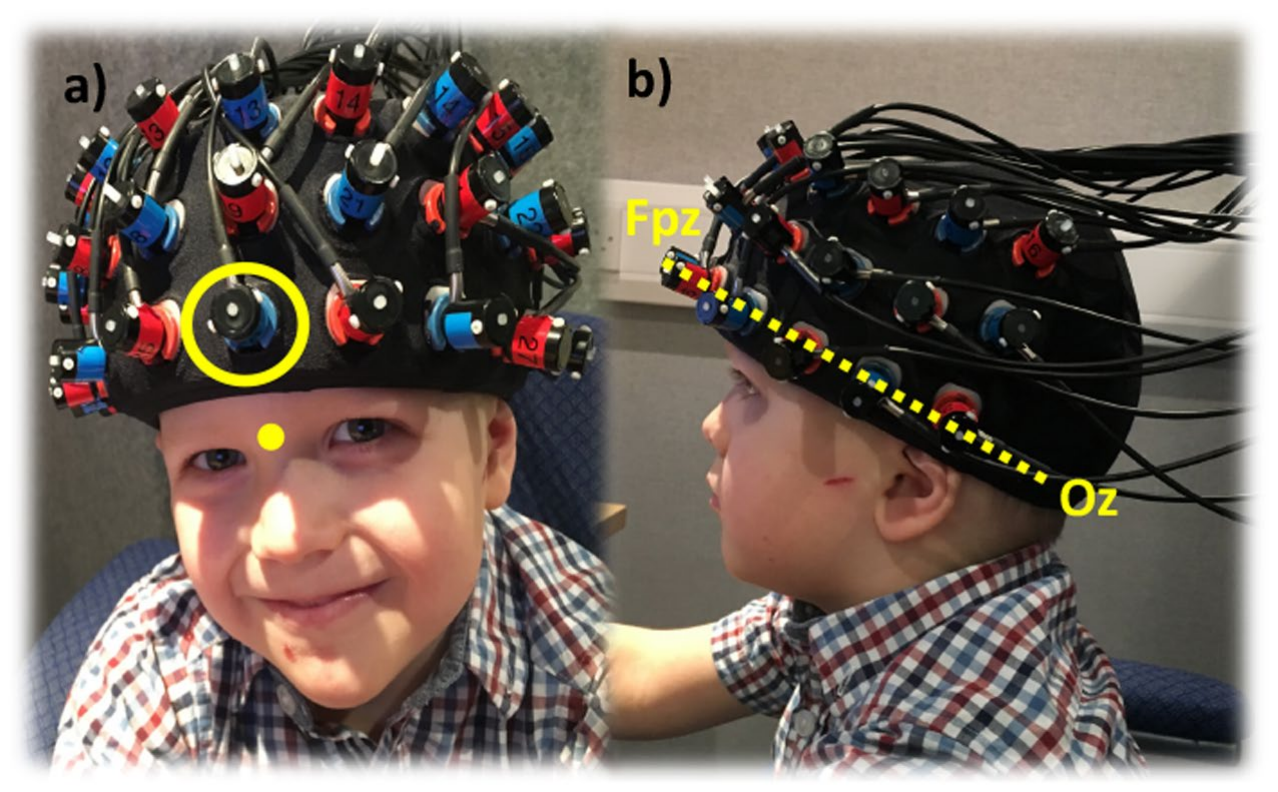
Placement of the fNIRS measurement array. **(a)** Photographic illustration of optode array placement with the emitter optode locations in red and detector optode locations in blue. The bottom centre optode (indicated by yellow circle) is placed at Fpz, which is located at 10% of the nasion to inion distance away from the nasion (indicated by the yellow dot); **(b)** The yellow dashed line indicates the trajectory from Fpz to Oz, which was used to guide placement of the lowermost row of optodes.

The international 10–20 system allowed correlation of defined external anatomical landmarks with underlying cortical areas (Jasper, 1958). The array was positioned on each participant’s forehead such that the emitter and detector optodes were centred horizontally at Fpz, a rationale commonly used in established fNIRS studies that have specifically examined EF-evoked cortical activation (Perlman et al., 2016; Smith et al., 2017). The bottom middle detector was carefully placed at location Fpz and the bottom row of optodes on each side aligned with the trajectory from Fpz and Oz (Figure 3). Although the array was constantly centred at Fpz, the inter-optode distance was fixed and hence the location of all other optodes was approximate and depended on the size and shape of each participant’s head. To evaluate variation in head size between individuals, the following measurements in centimetres were made on all participants: (1) head circumference, with the measurement taken along the trajectory of Fpz to Oz bilaterally; (2) the nasion to inion distance, and; (3) the distance between pre-auricular points.

**Figure 4:**
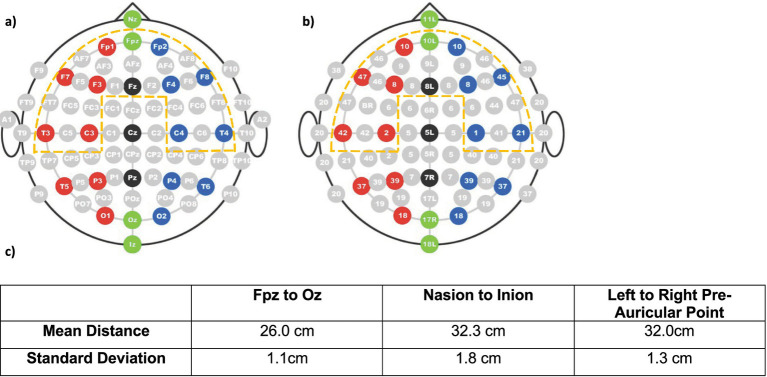
Typical coverage by the fNIRS optode array for all participants with respect to the international 10–20 system and corresponding Brodmann areas. The area of the brain typically covered by the optode array is indicated by the bold dashed line in relation to the positions of the 10–20 system in schematic **(a)** and the corresponding Brodmann areas in schematic **(b)**. The optode array typically spanned all cortical areas within the bold dashed line, hence good coverage of the prefrontal and superior temporal areas was achieved. The mean distance of specific measurements for 24 participants (NH children, *n* = 23; deaf child with CIs, *n* = 1) along with the standard deviations are shown in table **(c)**. The mean distances in this table, in combination with the landmarking system of the international 10–20 system, were used to devise the bold dashed line in both schematics **(a,b)**.

Specifically, we aimed to primarily measure cortical activation in the PFC and STC bilaterally. Since increased activation overlying Brodmann areas (BAs) 10 and 46 (located within the PFC) has been observed in multiple fNIRS studies examining responses to WM and IC tasks (Perlman et al., 2016; Smith et al., 2017), these BAs were targeted for the EF regions of interest (ROIs) bilaterally. Although the primary auditory cortex is located medially within the lateral sulcus (Penhune et al., 1996), secondary auditory regions important for speech processing are located more laterally within the STC (Friederici, 2011). These secondary auditory areas, which include BA 22 and 42, were targeted as auditory ROIs bilaterally. The definition of both EF and auditory ROIs was enabled by the international 10–20 system combined with measurements of participants’ head size. As the inter-optode distance within the array remained fixed, extrapolated measurements of the distance of each optode from defined landmarks of the 10–20 system made it possible to determine over which BA each fNIRS recording channel was approximately located in participants at the group level. Figure 4 shows the typical coverage of the fNIRS optode array in addition to mean distance of head measurements for the 24 participants that were included in the fNIRS data analysis (NH children, *n* = 23; deaf child with CIs, *n* = 1). Interestingly, the SD for all these measurements did not exceed the fixed inter-optode distance of 3 cm. The EF ROIs in the PFC were subsequently defined as right BA 10 (Ch#26, 36, 47), left BA 10 (Ch#27, 38, 48), right BA 46 (Ch#25, 35, 46) and left BA 46 (Ch#28, 39, 49). The ROIs in the STC were defined as the right and (Ch#32, 33, 43) left (Ch#41, 42, 52) auditory ROIs.

Once the position of the optode array was completed, a photograph was taken of the final placement for reference purposes. During testing, participants were instructed to remain still and keep head movements to a minimum to reduce motion artefacts in the recorded data.

#### 2.2.5 Analysis of fNIRS data

Analysis of the fNIRS data was performed in MATLAB (MathWorks, Natick, MA, USA) using functions provided in the HOMER2 package (Huppert et al., 2009) together with custom scripts. The analysis was performed in a similar manner to previous studies conducted in our laboratory (Anderson et al., 2017; Lawrence et al., 2018; Lawrence et al., 2020; Mushtaq et al., 2019, 2020; Wiggins et al., 2016; Wiggins and Hartley, 2015; Wijayasiri et al., 2017). The following steps were performed:

Exclusion of channels with poor signal quality: we used the scalp coupling index (SCI) to identify and exclude channels suffering from poor optode—scalp contact (Pollonini et al., 2014). We excluded channels with SCI < 0.3 chosen to exclude only the worst 5% of channels in our dataset. Experience from previous studies in our laboratory showed that excluding only the worst 5% of channels strikes an appropriate balance between the desire to include only high-quality channels in the analysis versus the risk of committing type II (false negative) statistical errors if the effective sample size is too heavily reduced due to extensive channel exclusions.

Conversion to optical density: the measured light intensity levels were converted to optical density using the HOMER2 *hmrIntensity2OD* function, a standard step in fNIRS data analysis (Huppert et al., 2009).

Motion-artefact correction: after the raw fNIRS intensity signals had been converted, a wavelet filtering technique was conducted using the HOMER2 *hmrMotionCorrelationWavelet* function, a technique described by Molavi and Dumont (2012). This enabled correction of motion artefact by eliminating outlying wavelength coefficients which are assumed to be artefacts by implementing a probability threshold. We excluded wavelet coefficients lying further than 0.719 times the interquartile range below the first or above the third quartiles. By avoiding the need to reject contaminated trials from the data and instead processing the signals to suppress artefacts, wavelet filtering can enhance data yield (Molavi and Dumont, 2012).

Bandpass filtering: the optical density signals were band-pass filtered between 0.02 and 0.5 Hz to attenuate low-frequency drift and cardiac oscillations.

Conversion to estimated changes in haemoglobin concentrations: optical density signals were then converted to estimated changes in the concentration of HbO and HbR through application of the modified Beer–Lambert Law (Huppert et al., 2009). A default value of 6 was used for the differential path-length factor at both wavelengths. Note that the continuous-wave fNIRS system used in the present study allows for the estimation only of relative changes in haemoglobin concentrations across conditions and not absolute concentrations.

Isolation of the functional haemodynamic response: we applied the haemodynamic modality separation (HMS) algorithm described by Yamada et al. (2012) to isolate the functional component of the haemodynamic signal and suppress systemic physiological interference (Yamada et al., 2012). This algorithm attempts to separate functional and systemic signals based on the assumption that the correlation between HbO and HbR will be different in each case. Although this approach does not accurately account for all statistical properties of the noise typically found in fNIRS data (Huppert, 2016), in previous studies we have found application of this algorithm to be beneficial to the detection of auditory cortical activation (Lawrence et al., 2018; Lawrence et al., 2020; Wiggins et al., 2016; Wijayasiri et al., 2017); in particular, application of the HMS algorithm was shown to substantially improve the test–retest reliability of auditory fNIRS measurements (Wiggins et al., 2016).

Quantification of response amplitude: to quantify the level of behavioural EF task-evoked cortical response, the pre-processed fNIRS signal was subjected to a general linear model (GLM) approach previously described in Wijayasiri et al. (2017) and Lawrence et al. (2018, 2020). The GLM was applied to the continuous data collected over the duration of the imaging session. The design matrix included a set of three regressors (corresponding to the canonical haemodynamic response plus its first two temporal derivatives) for each experimental condition within each behavioural task, plus a further set for the rest trials within each task. Each trial in each behavioural task was modelled as a short epoch corresponding to the actual duration stimulation for that trial. Within each condition, the canonical and temporal-derivative regressors were serially orthogonalized with respect to one another (Calhoun et al., 2004). Model estimation was performed using the two-stage ordinary least squares procedure described by Plichta et al. (2007), which incorporates a correction for serial correlation (Cochrane and Orcutt, 1949). The ‘derivative-boost’ technique (Calhoun et al., 2004) was used to estimate response amplitude: this technique calculates an amplitude value that is a function of both the canonical (nonderivative) and the derivative terms of the model; the resulting amplitude estimates are less affected by any systematic differences in latency or dispersion between conditions, compared to if the amplitude is estimated from the canonical term alone.

The beta weights of the canonical haemodynamic response function (HRF) were extracted at each measurement channel, for each stimulation condition within each behavioural task, and for all participants. The employed haemodynamic signal separation method (Yamada et al., 2012) assumes a fixed linear relationship between HbO and HbR in the functional response. Therefore, the results of all statistical analyses are identical regardless of whether conducted on the beta weights extracted for the HbO or HbR parameter. Only results corresponding to the beta estimates of the HbO parameter of the functional component are presented here. These beta weights were used to quantify the amplitude of behavioural EF task-evoked cortical activation and were subjected to further statistical analyses as specified in the relevant sections of this manuscript.

#### 2.2.6 Assessment of language performance

When originally designing the fNIRS experiment, a face-to-face assessment of language performance by a National Health Service (NHS) Speech and Language Therapist using the Preschool Language Scale—Fifth Edition (PLS-5^UK^®) was planned for all participants. Unfortunately, the COVID-19 pandemic commenced mid-way through the participant recruitment schedule. The unprecedented restrictions and demands of the pandemic on NHS clinical services prevented the NHS Speech and Language Therapists using the PLS-5^UK^® assessment for participants recruited after the onset of COVID-19. Due to the inability to continue with the PLS-5^UK^® assessment, the CCC-2® was administered to parents of all participants recruited to the fNIRS experiment after the pandemic commenced.

With respect to the PLS-5^UK^® assessment, this is an individually administered test that measures children’s receptive and expressive language skills (Zimmerman et al., 2014). It has been developed for use in children from birth to 7 years and 11 months and consists of two standardised scales which are auditory comprehension (AC) and expressive communication (EC). A total language score (TLS), a combination of the AC and EC score, may also be calculated. PLS-5^UK^® provides norm-referenced information due to being standardised on a UK population. It is therefore able to evaluate how a particular child is functioning in comparison to NH peers with both substantial reliability and validity (Zimmerman et al., 2014). The PLS-5^UK^® assessment has also been used by UK auditory implant programmes for ongoing manual monitoring of language development in CI recipients for many years after device insertion as part of their routine NHS care pathway.

The CCC-2® is a checklist that can be completed by a parent of a child aged 4 years and over who speaks in simple sentences (Bishop, 2003). The General Communication Composite (GCC) score calculated by the CCC-2® tool may be used to identify children who are likely to have clinically significant communication problems. Standardised GCC scores are based on normative data from a sample of children from the United Kingdom (UK). The CCC-2® has been demonstrated as a reliable and valid tool through data acquired from NH clinical samples (Bishop, 2013) and has also been shown to identify communication difficulties when administered to children using CIs (Ramirez-Inscoe and Moore, 2011).

### 2.3 Statistical analyses

#### 2.3.1 Behavioural data during fNIRS task

Bivariate linear regression analysis was employed to assess for any correlation between behavioural performance (d’prime task accuracy) on the Go/NoGo and N-back EF tasks and language ability in NH children (either PLS-5^UK^® TLS or CCC-2® GCC score). Twelve out of the 24 NH children performed a PLS-5^UK^® assessment prior to the onset of COVID-19, whereas the other 12 NH children were assessed with the CCC-2® tool. However, one child that was assessed with the PLS-5^UK^® tool refused to perform the N-back task. The mean age for children in the PLS-5^UK^® and CCC-2® groups was 5.2 (SD 0.89) and 5.0 (SD 0.71) years, respectively with no statistically significant difference in age between these two groups.

#### 2.3.2 fNIRS data

Our statistical analysis aimed to establish the relationship between language performance and the amplitude of behavioural EF task-evoked cortical activation in NH children measured with fNIRS on a channel-wise basis. One NH child refused to wear the fNIRS equipment whilst performing the Go/NoGo task and refused to perform the N-back task. Therefore, cortical activation data acquired from 23 NH children and one deaf child with CIs during the Go/NoGo and N-back tasks was subjected to analysis. With respect to the statistical analyses that examined the relationship between cortical correlates of EF and language performance, the 23 NH children were allocated to subgroups according to whether the PLS-5^UK^® (*n* = 11) or CCC-2® (*n* = 12) was performed. Bivariate correlation analysis was performed using the value for the difference in the beta weight between the EF and control condition, i.e., (NoGo vs. Go and 1 back vs. 0-back) and the PLS-5^UK^®/CCC-2® score.

To examine cortical responses during the behavioural Go/NoGo and N-back tasks, beta weights representing the amplitude of cortical activation for each condition within each task were extracted for each measurement channel across the optode array. For each condition within a task (i.e., the Go and NoGo conditions within the Go/NoGo task and the 0-back and 1-back conditions within the N-back task), single sample, one-tailed *t*-tests were conducted on the beta weights to detect measurement channels that displayed significant levels of cortical activation compared to rest. The same analysis was also performed to detect which measurement channels detected significantly increased cortical activation from the control to the EF condition (i.e., from the Go to the NoGo condition and from the 0-back to the 1-back condition). However, by testing for significant activation in all 52 individual measurement channels, multiple comparisons are performed and the risk of Type I error is increased. These multiple comparisons were accounted for by applying the false discovery rate (FDR method) across channels (Benjamini and Hochberg 1995). The original formulation of the FDR procedure, which assumes independence or slight positive dependency across tests, was used in line with recommendations for fNIRS data analysis (Singh and Dan, 2006). Statistical significance was assessed against an FDR-corrected threshold of *q* < 0.05.

For each condition within the Go/NoGo and N-back tasks, the time course of cortical activation within the predefined ROIs was plotted. This enabled visualisation of the behavioural EF-evoked haemodynamic responses to determine whether plausible, artefact-free, HRFs had been obtained in each task condition. For each measurement channel, the time course of HbO and HbR concentration changes were block-averaged using the HOMER2 hmrBlockAvg function (Huppert et al., 2009). For each task condition these were then averaged over the relevant measurement channels to produce separate time courses for each ROI.

Following the use of a GLM approach, the resultant beta weights representing the amplitude of cortical activation were averaged across the ROI measurement channels for each participant. To investigate cortical responses to the Go/NoGo and N-back tasks in NH children, beta weights for each condition were subjected to statistical testing using a Linear Mixed Model [LMM] (West et al., 2007). Analyses were conducted using IBM SPSS Statistics for Windows Version 26.0 software (IBM Corporation, Armonk, NY, USA). Unfortunately, the sample size for deaf children with CIs prevented statistical analysis with LMMs for this group. However, to investigate EF-evoked cortical activation in NH children during both the Go/NoGo and N-back tasks, beta weights were analysed separately for each ROI. Two LMMs were performed for each ROI. The first LMM included two fixed factors of ‘condition’ and ‘age group’ to estimate the fixed effect of task condition and age on behavioural EF-evoked cortical activation within each ROI. In addition, a ‘condition – age group’ interaction term was specified to understand whether an effect of condition on cortical activation differed between younger and older children. Age was represented as a continuous variable, but a median split of 4.9 years was used to allocate participants to either a ‘younger’ or ‘older’ age group so that the effect of age could be evaluated as a categorical variable within the model. To evaluate the effect of task performance (d’prime) on behavioural EF-evoked cortical activation, the covariate ‘performance (d’prime)’ was entered into the second LMM as a fixed effect. To account for variance related to age, ‘age’ was also specified as a fixed covariate in this LMM. In all LMMs, a random intercept for ‘participant’ was included with ‘scaled identity’ selected as the covariance type to account for the correlation between beta weights within a participant. A restricted maximum likelihood estimation method was adopted for all LMMs.

## 3 Results

### 3.1 fNIRS experiment

#### 3.1.1 The relationship between behavioural EF task performance and language ability

Performance accuracy (d’prime) on the Go/NoGo task was not associated with the PLS-5^UK^® TLS (*r* = −0.24, *p* = 0.45; *n* = 12) or CCC-2® GCC score (*T_b_* = 0.22, *p* = 0.34; *n* = 12) in NH children (both 2-tailed). Similar findings were observed for the N-back task with no association between d’prime and PLS-5^UK^® TLS (*r* = 0.08, *p* = 0.83; *n* = 11) or d’prime and CCC-2® GCC score (*T_b_* = 0.11, *p* = 0.63; *n* = 12) in NH children (both 2-tailed).

#### 3.1.2 Channel-wise analysis of relationships with behavioural EF

**Figure 5:**
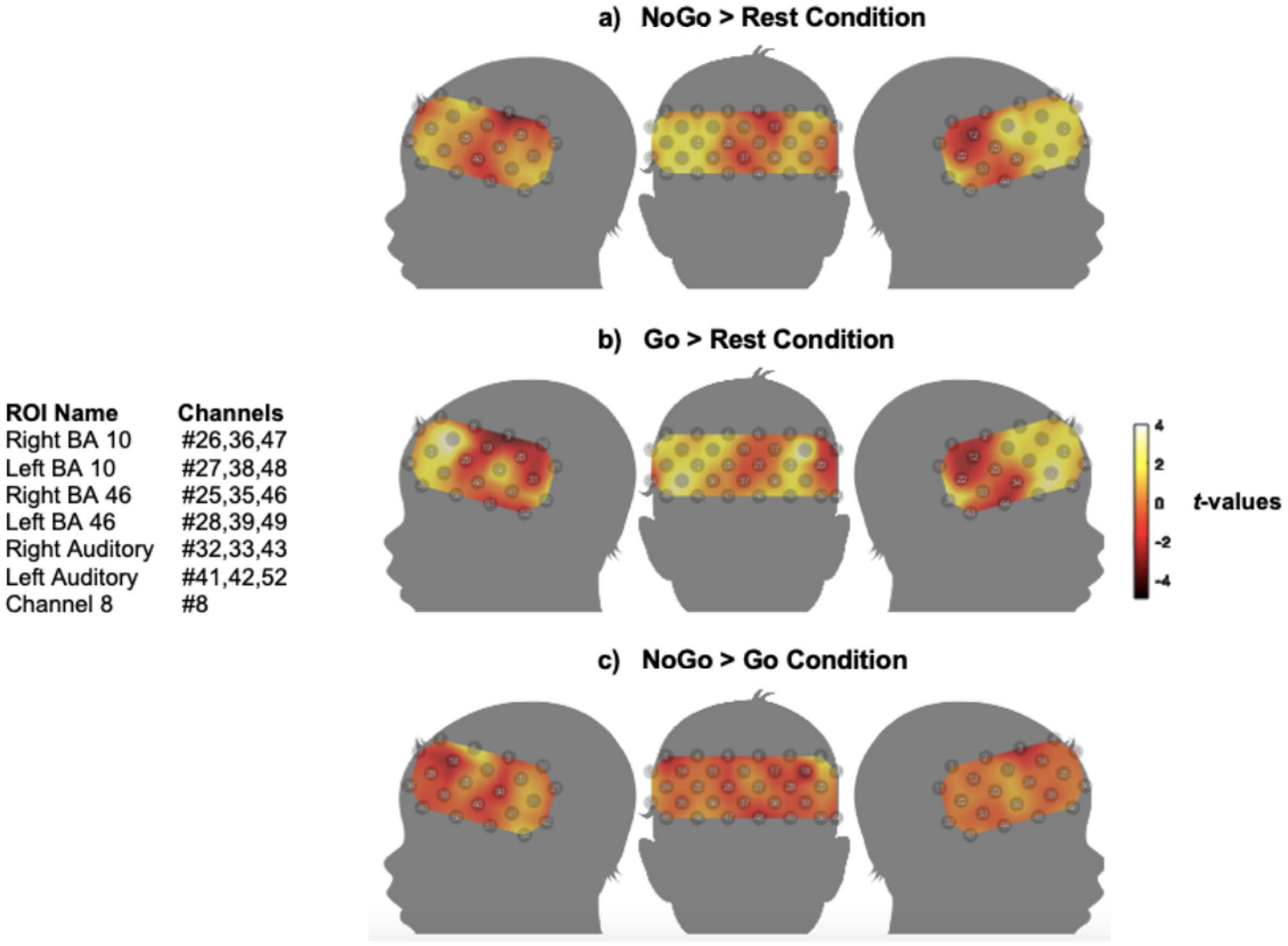
Group-level cortical activation maps for NH children during the Go/NoGo task. **(a)** NoGo condition > rest condition; **(b)** Go condition > rest condition; **(c)** NoGo > Go condition. Amplitude of cortical activation is colour coded by *t*-value. Although no significantly activated channels were identified, they were examined for with one-tailed *t*-tests (*q* < 0.05, FDR corrected). *n* = 23. Note the maps are interpolated from single-channel results and the overlay onto a paediatric template is for illustrative purposes only. N.B. *t*-value maps are uncorrected and for visualization only.

**Figure 6:**
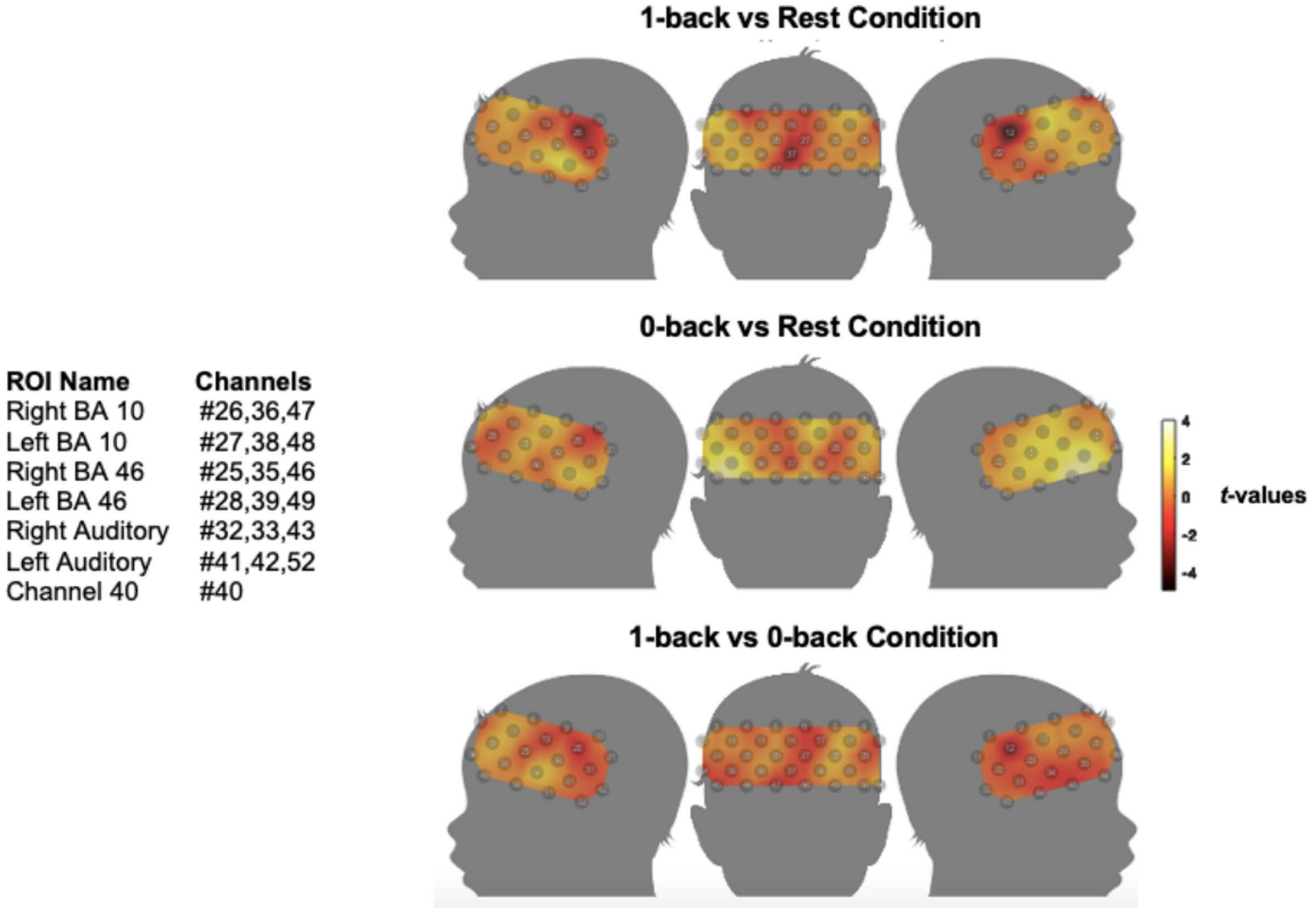
Group-level cortical activation maps for NH children during the N-back task. **(a)** 1-back condition > rest condition; **(b)** 0-back condition > rest condition; **(c)** 1-back > 0-back condition. Amplitude of cortical activation is colour coded by *t*-value. Although no significantly activated channels were identified, they were examined for with one-tailed *t*-tests (*q* < 0.05, FDR corrected). *n* = 23. The maps are interpolated from single-channel results and the overlay onto the paediatric template is for illustrative purposes only. N.B. *t*-value maps are uncorrected and for visualization only.

The results of the analysis for testing for systematic relationships between fNIRS response amplitude and behavioural EF tasks are shown in Figures 5, 6, which display group-level cortical activation maps for NH children for the Go/NoGo task and N-back tasks, respectively.

Figure 5 shows that after correction for multiple comparisons, no measurement channels were significantly activated across the group of NH children in either the NoGo (IC) or Go (control) task condition compared with rest (Figures 5a,b), or the NoGo vs. the Go condition (Figure 5c). However, increased cortical activation from the Go to the NoGo condition was apparent in channels overlying the left STC (Ch#41,42,52) and channel 8 (Ch#8); Figure 5c. The utilisation of mean head size measurements combined with the 10–20 system suggested that Ch#8 overlayed left BA 46. Although the predefined ‘left BA 46’ EF ROI (Ch#28,39,49) was also estimated to cover the region of the left lateral PFC, Ch#8 was measured to overlay BA 46 within a more posterior and dorsal area. It is commonly accepted in the literature that BA 46 is located within the dorsolateral prefrontal cortex (DLPFC) (Badre, 2008; Perlman et al., 2016; Smith et al., 2017), hence both the ‘left BA 46’ EF ROI and Ch#8 overlaid the left DLPFC.

Figure 6 demonstrates that no measurement channels were significantly activated across the group of NH children in either the 0-back control or 1-back WM condition compared with rest (Figures 6a,b), or the 1-back vs. the 0-back condition (Figure 6c). Despite not reaching significance, increased activation from the 0-back to the 1-back condition was apparent in channels overlying left BA 46 in the DLPFC (Ch#28,39,49) and channel 40 (Ch#40). Channel 40 was measured to overlay BA 44 within the left ventrolateral PFC (VLPFC).

#### 3.1.3 Response profiles in regions-of-interest

**Figure 7:**
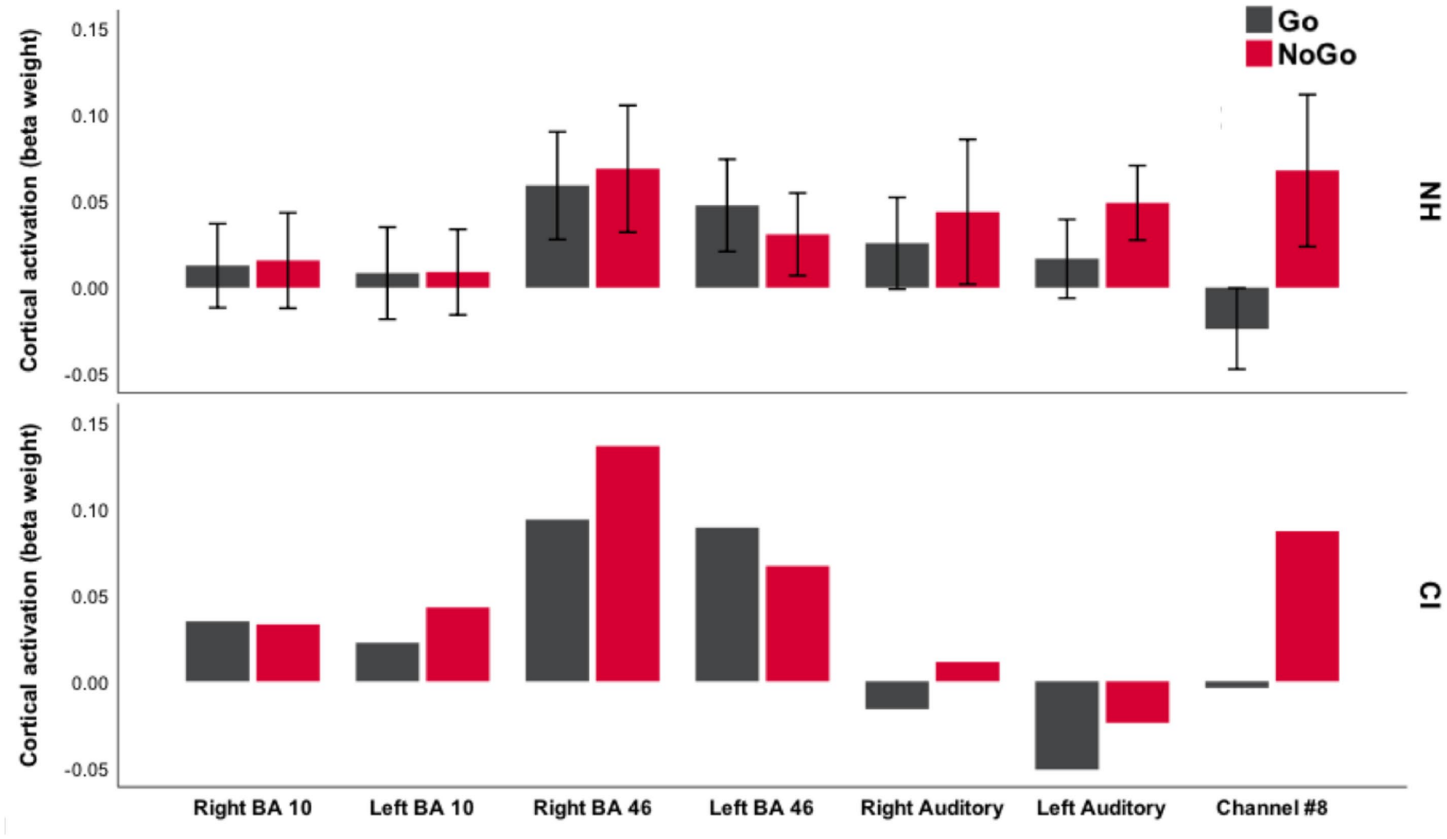
Bar chart of mean amplitude of cortical activation during the Go/NoGo task. This demonstrates beta weights for the NH children (mean for *n* = 23 participants) and one deaf child with CIs. Error bars show ±1 standard error of the mean (SEM).

**Figure 8:**
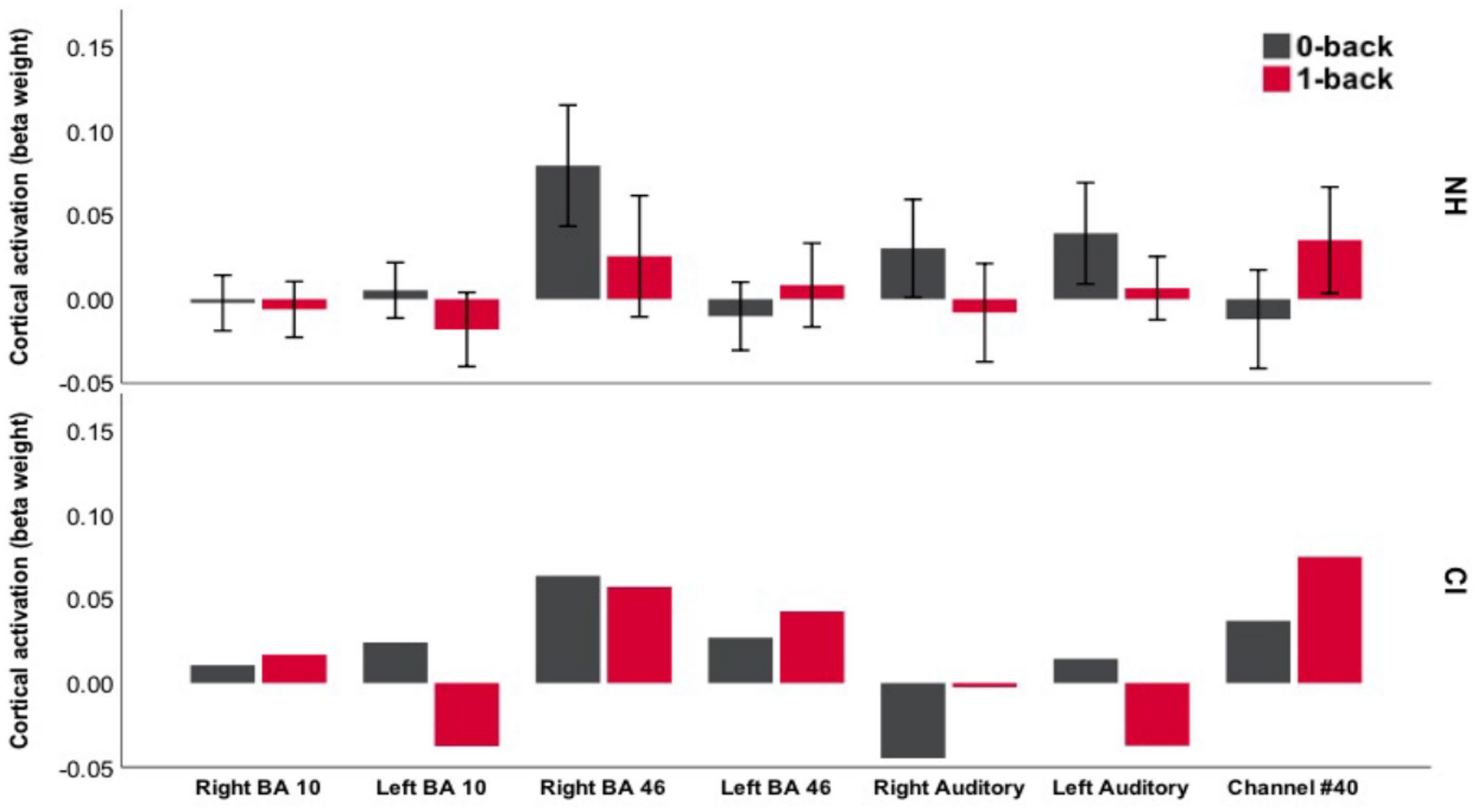
Bar chart of mean amplitude of cortical activation during the N-back task. This demonstrates beta weights for the NH children (mean for *n* = 23 participants) and one deaf child with CIs. Error bars show ±1 standard error of the mean (SEM).

To detail how fNIRS response amplitude varied during the IC and WM conditions in different parts of the brain, Figures 7, 8 plot the mean beta weight derived from each ROI during the EF and control conditions in the Go/NoGo and N-back tasks.

In NH children, increased activation was observed during the Go condition across most ROIs, albeit predominantly in bilateral BA 46 ROIs (Figure 7). Consistent with the cortical activation map, the beta weight substantially increased from the Go to the NoGo condition in Ch#8 and the left auditory ROI. Although extreme caution should be exercised when making comparisons, akin to NH children, cortical activation increased from the Go to the NoGo condition in Ch#8 for the deaf child with CIs. Conversely, a relative deactivation compared to rest was seen in the left auditory ROI of the deaf child with CIs during both the Go and NoGo conditions.

LMM analysis revealed a significant interaction between condition and age group (*F*_1,21_ = 6.44, *p* < 0.05) in the right BA 10 ROI which is commonly accepted to be part of the VLPFC (Perlman et al., 2016; Smith et al., 2017), with older children (≥4.9 years) displaying a significantly higher amplitude of cortical activation in the NoGo condition compared with Go condition (mean difference in beta weight = 0.064). After adjusting for main effects, there was a significant interaction between condition and age group in the left auditory ROI, with older children displaying greater cortical activation with increasing age during the NoGo condition compared with the Go condition (*β* = 0.095, *SE* = 0.03, *t* = 3.21, *p* < 0.05). In Ch#8, there was a significant main effect of condition (*F*_1,21_ = 5.23, *p* < 0.05) with a greater amplitude of cortical activation during the NoGo compared with the Go condition (mean difference 0.099). This effect appears to have been driven by age, as a significant interaction between condition and age group (*F*_1,21_ = 3.84, *p* < 0.05) was also observed, with younger children (<4.9 years) displaying significantly increased cortical activation in Ch#8 during the NoGo compared with Go condition (mean difference in beta weight 0.177). There was no significant main effect of Go/NoGo task performance (d’prime) on the amplitude of cortical activation across the ROIs.

With respect to the N-back task, increased activation during the 0-back control condition was apparent in the right BA 46 ROI and both auditory ROIs in NH children (Figure 8). Ch#40 detected an increased amplitude of cortical activation during the 1-back WM condition and a relative deactivation (compared to rest) during the 0-back condition. Akin to NH children, the deaf child with CIs displayed increased activation from the 0-back to the 1-back condition in Ch #40. Interestingly, deactivation compared with rest was observed in the right auditory ROI during both conditions and in the left auditory ROI during the 1-back condition for the deaf child with CIs. The only significant finding from LMM analysis was a main effect of task performance (*F*_1,20_ = 5.40, *p* < 0.05) on cortical activation detected by Ch#40, with increasing task performance (d’prime) associated with a greater amplitude of cortical activation during the 1-back condition (*β* = 0.074, *SE* = 0.03, *t* = 2.32, *p* < 0.05).

#### 3.1.4 The relationship between cortical correlates of behavioural EF and language ability

**Figure 9:**
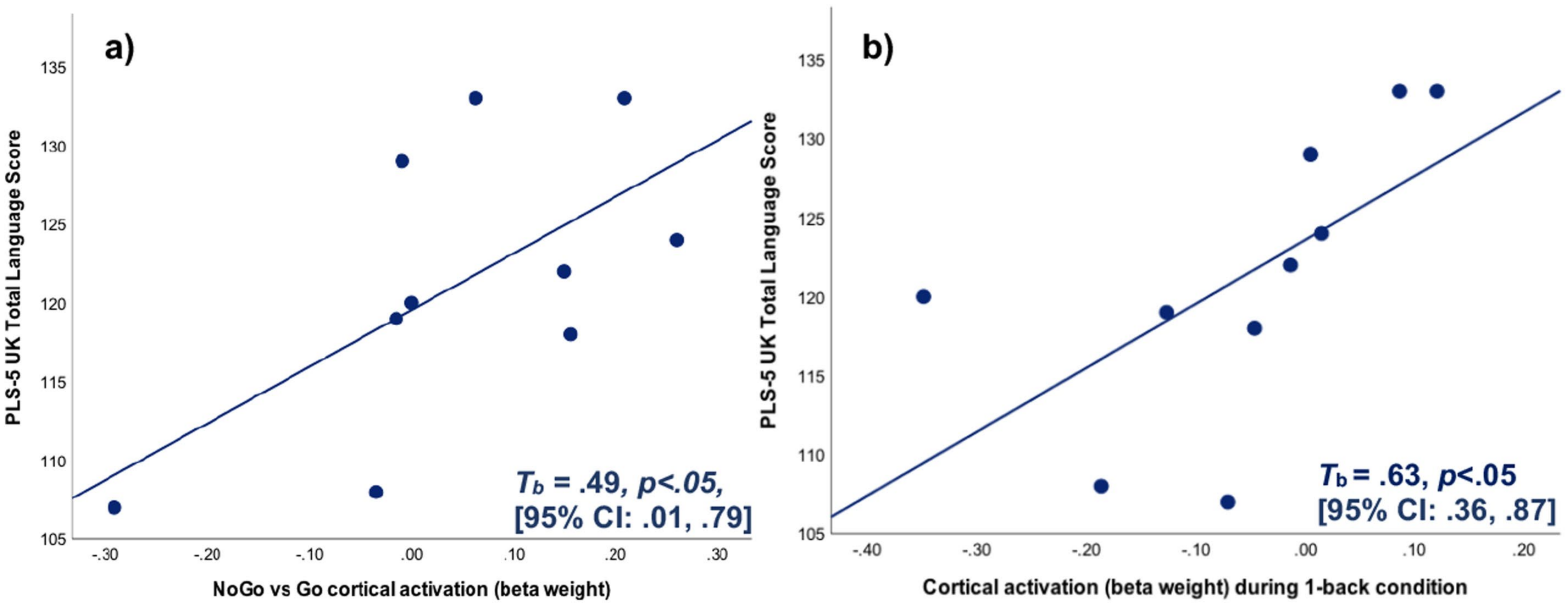
Scatterplots for **(a)** the beta weight measured for the NoGo vs Go condition and PLS-5^UK®^ total language score in channel #8 ROI in NH children (*n* = 10) and **(b)** the beta weight measured for the 1-back condition (vs rest) and PLS-5^UK^ total language score in the left BA 46 ROI in NH children (*n* = 11).

The amplitude of behavioural IC-task induced cortical activation in Ch#8 overlying left BA 46 during the NoGo vs. Go condition significantly correlated with the PLS-5^UK^® TLS (Figure 9a) in NH children, whereby increasing beta weight was associated with increased language scores (*T*_b_ = 0.49, *p* < 0.05, 2-tailed, 95% CI: 0.01, 0.79; *n* = 10). Since one NH child had Ch#8 excluded due to the absence of valid numerical values in the fNIRS raw chromophore concentration data, only 10 NH children were included in the bivariate correlation analysis for this ROI. In four of the 10 NH children, the measured beta weight was negative in value, signifying a lower amplitude of cortical activation (or greater relative deactivation) during the NoGo compared with Go condition. However, all 10 NH children had a PLS-5^UK^® TLS of >100, hence exhibited a language score within normal limits (a standard score of 100 represents the performance of the typical child of a given age, with scores between 85 and 115 corresponding to one SD below and above the mean). These results suggest that a lower beta weight value for the NoGo compared with Go condition in left BA 46 is not necessarily a neural correlate of poor language performance in NH children, rather an increasing beta weight value for the NoGo compared with Go condition (i.e., a greater amplitude of cortical activation or a lesser degree of relative deactivation) within this brain region is associated with higher language scores in children that are already performing within clinically normal limits.

In terms of WM, the amplitude of behavioural task-evoked cortical activation in the left BA 46 ROI during the 1-back condition when compared with rest was significantly associated with PLS-5^UK^® TLS performance (*T*_b_ = 0.63, *p* < 0.05, 2-tailed, 95% CI: 0.36, 0.87; *n* = 11), see Figure 9b. Six participants exhibited a relative deactivation during the 1-back WM condition compared with rest, however akin to the Go/NoGo task, all these participants had PLS-5^UK^® scores within normal limits suggesting that WM task-evoked cortical deactivation (albeit relative to rest in this case) is not necessarily indicative of deficient language performance.

The correlation between PLS-5^UK^® performance and behavioural EF-evoked cortical activation in all remaining ROIs (for both the Go/NoGo and N-back task) was statistically non-significant (*n* = 11). Interestingly, performance on the CCC-2® parent-reported measure was not associated with the amplitude of behavioural EF-evoked cortical activation measured across all ROIs for each of the IC and WM tasks (*n* = 12). In terms of analysing cortical correlates of behavioural EF and their association with language performance, it is arguably more appropriate to compare the amplitude of activation measured during the EF condition (NoGo and 1-back conditions) with that acquired during the control condition (Go and 0-back), rather than with rest. Nonetheless, regardless of the comparison made, all statistically significant associations between behavioural EF-evoked cortical activation and language performance related to cortical correlates acquired from the left BA 46 ROI overlying the left DLPFC.

## 4 Discussion

This study provides the first known investigation of the relationship between behavioural EF-evoked cortical correlates of and language ability. A significant association between the amplitude of visual IC and WM task-evoked cortical activation overlying the left DLPFC and language performance was observed in NH children, supporting the theory that top-down cognitive processes facilitate language acquisition. Furthermore, our results appear to be the first to demonstrate that fNIRS is capable of successfully measuring responses, specifically to behavioural EF, over bilateral prefrontal and superior temporal regions in a paediatric CI recipient.

### 4.1 The association between behavioural measures of EF and language

In the present study, no significant association was observed between language ability and behavioural performance on either the visual IC Go/NoGo task or the WM N-back task in NH children. This is inconsistent with a report in the literature that describes a significant correlation between performance on visual IC and visuospatial WM N-back tasks and language skills in typically-developing children (Kronenberger et al., 2014b). This study recruited substantially more participants than our experiment, hence our findings may relate to our relatively small sample size.

### 4.2 Cortical correlates of behavioural executive function

Numerous studies have demonstrated the capability of fNIRS to acquire cortical correlates of behavioural EF in NH children. However, unlike the present study, the majority of these studies have examined EF-evoked cortical activation over frontal and/or parietal regions without consideration of STC (Baird et al., 2002; Buss et al., 2014; Gu et al., 2018; Mehnert et al., 2013; Perlman et al., 2016; Smith et al., 2017). Although statistical testing with one-tailed *t*-tests failed to identify any significantly activated channels in NH children, subsequent LMM analysis indicated significant EF-evoked cortical activation in certain channels, specifically a significant main effect of condition for the Go/NoGo task in Ch#8 overlying left BA 46. These differences potentially relate to the necessary adjustment for the multiple comparisons following the one-tailed *t*-tests, which may have tipped any findings into insignificance. Speculatively, a larger sample size may have yielded different conclusions.

The bilateral DLPFC is reportedly the main correlate of the central executive, which according to Baddeley and Hitch’s model of WM, is hypothesised to exert overall attentional control over visuospatial and phonological WM processes (Baddeley and Hitch, 1974). In NH children and the deaf child with CIs, substantial activation was observed overlying the bilateral DLPFC during both control conditions of the Go/NoGo and N-back tasks, a finding consistent with existing fNIRS studies for NH children (Mehnert et al., 2013; Smith et al., 2017). This suggests that each control condition within each EF task demanded an element of attention and WM.

With respect to the Go/NoGo task, the statistically significant observation that IC task-evoked cortical activation increases with age within the right VLPFC of NH children and decreases with age in the left DLPFC aligns with reports from fNIRS, functional magnetic resonance imaging (fMRI) and magnetoencephalography (MEG) studies (Durston et al., 2006; Mehnert et al., 2013; Vara et al., 2014). Furthermore, consistent with existing fNIRS (Smith et al., 2017) and fMRI (Braver et al., 1997; Cohen et al., 1997) studies, substantially increased WM-induced activation overlying left BA 44 of NH children and the deaf child with CIs was observed during the N-back task. Specifically, BA 44 is located within the left inferior frontal gyrus (LIFG), a cortical region already identified as a possible neural correlate of effortful listening (White and Langdon, 2021; Wild et al., 2012). However, a previous adult fNIRS study from our laboratory demonstrated that the LIFG only exhibited an elevated response to degraded speech when attention was directed towards the stimulus (Wijayasiri et al., 2017). An increased amplitude of WM-evoked activation overlying the left BA 44/LIFG may be more indicative of the amount of attention paid to the task, since our current experiments used visual and not auditory based tasks. This theory is supported by the finding that a significantly greater amplitude of activation overlying left BA 44 was observed with increasing task performance accuracy during the N-back task. Conversely, there was no significant correlation between Go/NoGo task performance and the amplitude of activation across the ROIs. The observation that the deaf child with CIs exhibited a similarly increased amplitude of visual IC and WM task-evoked cortical within prefrontal regions when compared with their NH peers, yet a lower amplitude of such activation within the STC may relate to the process of neuronal decoupling of prefrontal and auditory areas in the auditory-deprived brain (Kral and Sharma, 2012). Deficient connectivity between these brain regions may be particularly problematic for verbal WM processing, as evidence suggests that such processing mechanisms are dependent upon both these cortical areas (Baddeley, 2003; White and Langdon, 2021). Verbal WM is reported to have the strongest predictive relationship with language ability in paediatric CI users (Kronenberger et al., 2014b; Kronenberger et al., 2020), which raises the question as to whether the strength of connectivity between prefrontal and superior temporal regions may contribute to the observed variance in CI language outcome. However, findings in this current study need to be interpreted with caution since the CI participant was 4.3 years of age, whereas the average age of the NH participants was 5.3 years. Hence, this one-year age gap may instead potentially represent substantial differences in neurodevelopmental immaturity rather than auditory deprivation per se.

### 4.3 The relationship between behavioural EF-evoked cortical activation and language performance

Convergent evidence from previous neuroimaging studies has shown that listening under adverse conditions modulates input from the PFC, suggesting that top-down EF processes regulate listening effort (Du et al., 2016; Mattys et al., 2009; Peelle, 2018). In a recent adult fNIRS study, a distinct pattern of activation within frontal and left temporo-parietal brain regions was observed whilst listeners were exposed to increasingly adverse listening conditions (White and Langdon, 2021). The least challenging conditions were found to recruit the LIFG, whereas increasingly difficult conditions (simulating CI listening) were observed to sequentially activate the left inferior parietal lobe (IPL) and ultimately the left DLPFC and right BA 10 regions. Since both the left IPL and left DLPFC areas are implicated in verbal WM processing (Baddeley, 2003) and the right BA 10 region in auditory attention processing (Medalla and Barbas, 2014) these findings suggest that difficult listening conditions are particularly reliant on higher-order attention and verbal WM mechanisms. In the current study, we observed a statistically significant association between performance on the PLS-5^UK^® assessment and visual IC and WM task-evoked activation within the left BA 46 ROI overlying the left DLPFC in NH children. Although no significant correlation was observed between behavioural EF-evoked cortical activation overlying right BA 10 and language ability, statistical analysis did reveal the level of brain activation in this area to significantly increase with age suggesting that these specific auditory attention processes may become more robust with chronological age. Our current findings suggest that children who dedicate greater attentional control to visual EF tasks, and more actively recruit the central executive within the DLPFC, are perhaps also more likely to allocate such effortful resources to other tasks such as those involving listening and language acquisition. This theory is supported by the observation in the study by White and Langdon (2021), where it is reported that listening effort was driven by a voluntary allocation of attentional resources.

Future work aimed at establishing whether EF-evoked cortical activation within the left DLPFC of deaf children is significantly associated with language outcome, as was observed for NH children in the current study, would be incredibly informative. There is already evidence to suggest that recruitment of the left DLPFC may be predictive of future language performance in paediatric CI recipients. A PET study which examined resting-state cortical activity in severe-to-profoundly deaf children (aged 1–11 years) prior to cochlear implantation demonstrated that individuals with good speech understanding 3 years post-implantation exhibited enhanced metabolic activity in the left DLPFC compared with those who had poor speech perception (Lee et al., 2007). Interestingly, a recent imaging study using high-density diffuse optical tomography (HD-DOT) that recruited deaf-implanted and NH adults showed that whilst listening to spoken words in quiet, listeners with CIs showed greater activity in the left PFC than NH controls (Sherafati et al., 2022).

### 4.4 Limitations and potential clinical impact

A limitation of the present study is that our results, along with the findings of other fNIRS studies, are yet to be applied and interpreted at the individual level. Advances in the fNIRS testing protocols and data analysis are therefore required to efficiently report their significance at the individual level and furthermore allow for this to occur real-time in the clinical setting. Other CI compatible imaging techniques such as EEG offer improved temporal resolution (Lloyd-Fox et al., 2010) and potentially reduced cost when compared with fNIRS, yet have drawbacks such as being more sensitive to head movements, which may be particularly problematic in children. Nonetheless, the development of specific high-density fNIRS systems which employ multi-distance source-detector separations are leading to an ever increasing overall accuracy of fNIRS imaging (Shin et al., 2017).

Caution should also be exercised over our findings that relate to the association between cortical correlates of behavioural EF and language ability due to the exploratory nature of these analyses given the small sample size of NH subjects. Given that channel-wise analyses showed no significant EF evoked-cortical activation after FDR correction whilst the ROI analyses did, it must be acknowledged that the ROI findings in this current study are hypothesis-driven based on prior literature and personal laboratory experience (Lawrence et al., 2020, 2018; Mushtaq et al., 2020, 2019), and not post-hoc selection. Additionally, analyses were exploratory and the observed statistically significant results may have arisen simply by chance since the statistics were not corrected for multiple comparisons.

Although behavioural EF-evoked activation was significantly associated with the PLS-5^UK^®, it was not associated with the CCC-2® parent-reported measure of language performance. This may relate to reporter bias associated with parent-reported measures. Conversely, it could be argued that an evaluation of a child’s language skills by a parent who observes the child daily provides a more accurate reflection of their language ability than a one-off clinical assessment by a healthcare professional. A further limitation is the cross-sectional design of the experiments which does not allow causation to be inferred. Observational, statistically powered longitudinal studies involving both NH children and paediatric CI users would provide a more accurate investigation into the relationship between cortical correlates of behavioural EF in both the normally hearing and auditory-deprived brain and language outcome. The wide age range for subjects in this study also potentially limits interpretability of the findings. Although the age range was selected with the aim of analysing the effect of age and cortical maturity on EF-evoked cortical activation, the lack of repeated measurements in the same individuals at different ages further precludes the ability to address longitudinal patterns. Lastly, in terms of limitations, the dissociation between behavioural performance (d’prime) and cortical activation suggests potential ceiling effects in the tasks for the age range in this current study. Although reaction time data was not analysed in this study, such data would be useful to evaluate the concept of neural effort and potentially reveal associations absent in the accuracy measures within this study.

With respect to potential clinical impact of our study findings, cortical activation elicited by EF behaviours may offer important information about the attention and effort that an individual dedicates to language acquisition, which may subsequently help explain the variability in language observed amongst children with and without CIs. Future applications of fNIRS technology could help to identify individuals with deficits in EF and subsequently facilitate appropriate intervention at an earlier stage in a child’s life. Since evidence from numerous studies indicates that cognitive training is effective in NH children (Jaeggi et al., 2011; Karbach and Kray, 2009; Thorell et al., 2009) and paediatric CI users (Kronenberger et al., 2011), improving EF has the potential to have a positive clinical impact on language outcome in children with and without a hearing loss.

## Data Availability

The original contributions presented in the study are included in the article/supplementary material, further inquiries can be directed to the corresponding author.
